# Targeting Hepatocellular Carcinoma Growth: Haprolid’s Inhibition of AKT Signaling Through DExH-Box Helicase 9 Downregulation

**DOI:** 10.3390/cancers17030443

**Published:** 2025-01-28

**Authors:** Jun Xing, Xiaoxi Feng, Rutong Zhang, Kaiguang Zhang

**Affiliations:** Department of Digestive Disease, The First Affiliated Hospital of USTC, Division of Life Sciences and Medicine, University of Science and Technology of China, Hefei 230001, China; xingjunjoy@hotmail.com (J.X.);

**Keywords:** haprolid, hepatocellular carcinoma, DHX9, AKT, iTRAQ

## Abstract

**Objective:** Haprolid, a novel compound extracted from Myxobacterium, has been proven to possess selective toxicity towards various tumor cells, effectively inhibiting the growth of hepatocellular carcinoma (HCC). However, the underlying molecular mechanism remains unclear. **Methods:** To identify differentially expressed proteins (DEPs), isobaric tags for relative and absolute quantitation (iTRAQ) were employed. The clinical significance of DExH-Box Helicase 9 (DHX9) was determined using tissue microarrays in HCC patients. Changes in protein expression were detected using Western blotting, qPCR, and immunohistochemistry. Cell proliferation was evaluated using CCK-8 and crystal violet staining. Cell apoptosis was assessed using Alexa Fluor 647 Annexin V. Xenograft tumor experiments were conducted in animals. **Results:** iTRAQ screening identified DHX9 as a DEP. DHX9 was discovered to be highly expressed in HCC tissues, correlating with poor prognosis in patients. Haprolid downregulated DHX9 expression, while knockdown of DHX9 suppressed HCC cell proliferation and migration and promoted apoptosis. Meanwhile, overexpression of DHX9 mitigated the inhibitory effect of Haprolid on HCC cells. Knockdown of DHX9 inhibited the AKT signaling pathway, and SC79 reversed the inhibitory effect of DHX9 knockdown on HCC cells. Xenograft experiments confirmed that the knockdown of DHX9 inhibited HCC growth, while the overexpression of DHX9 attenuated the inhibitory effect of Haprolid on HCC growth. **Conclusions:** Haprolid inhibits the AKT signaling pathway by downregulating DHX9, ultimately suppressing HCC growth. This finding opens up new avenues for targeted HCC therapy.

## Simple Summary

Haprolid, a novel compound derived from Myxobacterium, has demonstrated selective toxicity against various tumor cells and effectively inhibits the growth of hepatocellular carcinoma. However, its molecular mechanism remains unclear. This study identified DExH-Box Helicase 9 as a key protein through proteomics screening, revealing its high expression in hepatocellular carcinoma tissues and its correlation with poor patient prognosis. Haprolid downregulates DExH-Box Helicase 9 expression, suppressing cell proliferation and migration while promoting apoptosis. Overexpression of DExH-Box Helicase 9 mitigates Haprolid’s inhibitory effects. Further experiments showed that DExH-Box Helicase 9 regulates the AKT signaling pathway, and animal studies confirmed that DExH-Box Helicase 9 knockdown inhibits hepatocellular carcinoma growth, whereas its overexpression reduces Haprolid’s efficacy. These findings highlight Haprolid’s mechanism of action through DExH-Box Helicase 9 and AKT signaling, offering new insights for targeted hepatocellular carcinoma therapy.

## 1. Introduction

Primary liver cancer (PLC), one of the most prevalent malignant tumors worldwide, has long been identified as one of the primary causes of death worldwide. According to GLOBOCAN2020 statistics, approximately 906,000 new cases arise annually worldwide. These figures suggest that liver cancer ranks as the sixth most frequent and third deadliest malignant tumor [1]. Owing to the covert onset of hepatocellular carcinoma (HCC), over half of diagnosed patients present with intermediate to advanced disease stages. Although systemic antitumor treatments, such as tyrosine kinase inhibitions, immune checkpoint inhibitors, and monoclonal antibody treatments, have exhibited significant advancements [2,3], the overall five-year survival rate for patients is less than 20%. Hence, the exploration of more efficacious pharmacological interventions for HCC is of critical importance.

Isolated from *Byssovorax cruenta* in 2000, Haprolid is a novel polyketide–peptide hybrid small molecule with a unique macrolide structure comprising four modified amino acids as well as fragments from polyketides present. It is a white powder with the molecular formula C_38_H_58_N_4_O_7_ [4]. Through high-throughput screening of numerous bacterial extracts, Haprolid has demonstrated potent antitumor effects, particularly against HCC cell lines [4]. Notably, Haprolid exhibits selective cytotoxicity, and has been demonstrated to impede HCC growth significantly in in vitro and in vivo settings [5]. Moreover, Haprolid inhibits the proliferation, invasion, and migration of HCC cells. It also causes cycle arrest and promotes apoptosis in Hep3B cells [5]. Despite its evident therapeutic potential for HCC, the precise mechanism of action remains to be elucidated.

DExH-Box Helicase 9 (DHX9) is an adenosine triphosphate (ATP)-dependent enzyme that unwinds double-stranded RNA and DNA–RNA complexes. It is overexpressed in various tumor types, including lung, cervical, colorectal, and liver cancer [6,7]. In HCC, elevated DHX9 levels have been correlated with tumor node metastasis (TNM) stage, vascular invasion, and metastasis, serving as independent factors for poor prognosis [8]. Knockdown of DHX9 significantly reduces tumor growth, while long-term suppression in adult mice is tolerated well, making DHX9 an excellent target for cancer treatment [9]. Our study observed DHX9 downregulation in Hep3B cells following Haprolid treatment, as detected by isobaric tags for relative and absolute quantitation (iTRAQ) proteomic sequencing. This finding led us to speculate that DHX9 may be instrumental in mediating the inhibitory effects of Haprolid on HCC growth.

Accordingly, the objective of our study was to investigate the role of DHX9 in the inhibition of HCC growth by Haprolid and to further explore the molecular mechanisms underlying its depth.

## 2. Materials and Methods

### 2.1. Cells Culture

Hep3B, Huh7, and HepG2 cells were procured from Shanghai Fuheng Biotechnology Company. The cells were cultured in Dulbecco’s Modified Eagle Medium (DMEM) (Gibco, Waltham, MA, USA) supplemented with 5% FBS (Gibco), 5% NBCS (Gibco), and 1% antibiotic solution containing streptomycin and penicillin (Beyotime Biotechnology, Shanghai, China) at 37 °C in a humidified incubator containing 5% CO_2_.

### 2.2. iTRAQ

Hep3B cells were treated with 6 µg/mL Haprolid for 96 h, whereas the control group was treated with DMSO. Each treatment condition was replicated three times. After treatment, the samples were forwarded to Wuhan Jinkairui Bioengineering Company (Wuhan, China) for iTRAQ analysis.

### 2.3. Prognostic Analysis and Tissue Microarray Arrays

Tissue microarrays, comprising 75 pairs of HCC and paraneoplastic tissues, were generously provided by Shanghai Outdo Biotech Co., Ltd. (Shanghai, China). Immunohistochemical staining for DHX9 (Abcam, Boston, MA, USA; ab183731) was performed using tissue microarrays. Notably, one case of HCC tissue and two cases of paraneoplastic tissue were unavailable, resulting in a total of 74 HCC tissues and 73 paraneoplastic tissues for analysis. Staining intensity and positivity were evaluated by a pathologist. The grading criteria were as follows: (1) Staining intensity score: 0 for colorless, 1 for pale yellow, 2 for tan, and 3 for brown. (2) Scoring of staining positivity: 0 for negative, 1 for 1–25%, 2 for 26–50%, 3 for 51–75%, and 4 for 76–100%. The product of the staining intensity and staining positivity scores yielded the total score for categorization. Note that tissues scoring 8 or above have high DHX9 expression, whereas those with a score less than 8 are considered to have low DHX9 expression. In addition, the Kaplan–Meier Plotter website was used to assess the prognostic association between DHX9 expression and liver cancer.

### 2.4. Lentiviral Transfection and Drug Treatment

The knockdown plasmids for DHX9 were constructed by inserting the following small hairpin RNA (shRNA) sequences into the Plko.1 vector. The pLVX-Flag-DHX9 construct was created by inserting the Flag-tagged DHX9 sequence into the pLVX vector (Clontech, San Jose, CA, USA). Cells were selected using 1 µg/mL puromycin (Sigma, St. Louis, MO, USA) or 500 µg/mL G418 (Sigma) for 3-7 days to isolate stably transfected cell lines.

shDHX9-1: CCGGGGCTTTCGTTTAATACAATAGCTCGAGCTATTGTATTAAACGAAAGCCTTTTT

shDHX9-2: CCGGTCGAGGAATCAGTCATGTAATCTCGAGATTACATGACTGATTCCTCGATTTTT

Chromatographic techniques were used to extract Haprolid from the culture broth of *Byssovorax cruenta* [4]. Haprolid was dissolved in dimethyl sulfoxide (DMSO) (Sangon Biotech, Shanghai, China), and a stock solution was configured to be 10 mg/mL. Huh7 and Hep3B cells were treated with 6 µg/mL Haprolid for 96 h and used for subsequent experiments. Equal volumes of DMSO were used as the control. This concentration was referenced from Xing Jun et al. [5]. SC79, an AKT activator, was used to activate the AKT signaling pathway by treating cells at a concentration of 8 or 12 µg/mL for 48 h. For AKT inhibition, MK2206-2HCl (Sigma-Aldrich) was applied at a concentration of 5 µmol/L for 96 h.

### 2.5. Cell Counting Kit-8 Assay (CCK8)

Cell proliferation was assessed using Cell Counting Kit-8 (Topscience, Shanghai, China). Cells were seeded in 96-well plates, treated accordingly, and incubated with the CCK8 solution. After a 3 h incubation, the absorbance was measured at 450 nm.

### 2.6. Crystal Violet Staining

Cellular proliferative capacity and cytotoxicity were evaluated using crystal violet staining. Following treatment, 4% paraformaldehyde (4% PFA) was used to fix the cells for 30 min, followed by 10 min staining using a 0.2% solution of crystal violet (BBI life sciences, Shanghai, China). Unbound crystal violet was removed using phosphate-buffered saline (PBS) before photographing stained cells.

### 2.7. Apoptosis Assay

Live and dead cells were harvested and stained for 30 min with Alexa Fluor 647 Annexin V (BioLegend, San Diego, CA, USA) under light-protected conditions. Prior to flow cytometric analysis, DAPI (Sigma) was added to the samples. Apoptotic cells were quantified using a flow cytometer (Beckman CytoFLEX, Brea, CA, USA), and FlowJo (version 10.8.1) was used to analyze the data.

### 2.8. Transwell Assays

Transwell chambers (Corning, Corning, NY, USA) with pores of 8.0 µm were utilized to test cell migration capabilities. Treated cells were cultured in the upper chamber with 200 µL of 1% FBS medium, whereas 600 µL of 10% FBS medium was used as a chemoattractant in the lower chamber. After 24 h of incubation, 4% PFA was used to fix the chambers, and 0.1% crystal violet solution was used to stain. Note that excess crystal violet was rinsed away using PBS. Meanwhile, non-migratory cells in the upper chamber were carefully removed using a cotton swab. At the same time, migration was assessed by capturing images at 200× magnification using an Olympus microscope (Olympus, Tokyo, Japan).

### 2.9. Wound-Healing Scratch Assay

Treated cells were cultured in a 6-well plate. When the cells were fully grown, a straight wound was made with a 200 µL pipet tip. After gently washing the cells twice with PBS to remove detached cells, images of the wound were captured at 40× magnification using an Olympus microscope at specified time points (0, 24, and 48 h post-wounding). The extent of wound closure was quantitatively analyzed using ImageJ software (Version 2.1.0).

### 2.10. qRT-PCR

Total cellular RNA was isolated using TransGen Total RNA Isolation Reagent (Trizol) (TransGen, Beijing, China, ET101-01). RNA was reverse-transcribed to cDNA using the HiScript II 1st Strand cDNA Synthesis Kit (Vazyme, Nanjing, China, R212-02). qRT-PCR was conducted using the AceQ qPCR SYBR Green Master Mix (Vazyme, Q111-02). Data were collected using PikoReal software (version 2.2). The primers used in the qRT-PCR were as follows: DHX9:5′-CAG GAG AGA GAG TTA CTG CCT-3′ (forward) and 5′-CTC TGC TGC TCG GTC ATT CTG-3′ (reverse). All primers were synthesized by Sangon Biotech.

### 2.11. Protein Extraction and Western Blotting

Cells and tissues were lysed in a buffer containing 50 mM Tris-HCl, 150 mM NaCl, 50 mM NaF, 1 mM EDTA, 1 mM Na_2_P_2_O_4_, 1 mM Na_3_VO_4_, 1 mM PMSF, and cocktail (Sigma). Proteins were subsequently separated via SDS-PAGE and electro-transferred onto 0.45 µm polyvinylidene fluoride membranes (Millipore, Burlington, MA, USA). The membranes were blocked with 5% non-fat milk for 1 h at room temperature. Primary antibody incubation was performed overnight at 4 °C, followed by 1h incubation with the appropriate secondary antibody at room temperature. The following antibodies were used in this study: DHX9 (Abcam; ab26271), pAKT-Ser473 (CST, Danvers, MA, USA; 9271S), pAKT-Thr308 (CST; 2965S), AKT (CST; 4685S), Actin (Proteintect, Rosemont, IL, USA; 66009-1-Ig), and FLAG (66008-4-Ig).

### 2.12. Immunohistochemical Staining

Tumor tissue was fixed for paraffin embedding and subsequently cut into 4 µm tissue sections. The sections were incubated overnight at 4 °C with the primary antibodies, followed by a 1 h incubation with the appropriate secondary antibodies at room temperature. Staining was performed using 3,3′-diaminobenzidine and counterstained with hematoxylin. Photomicrographs were obtained using a microscope. Two independent pathologists blindly assessed immunostaining positivity. Five random fields were selected from each slide for examination. The antibodies used in this study included DHX9 (Abcam; ab183731) and Ki-67 (Abcam; ab16667).

### 2.13. Animal Experiments

Shanghai Slac Laboratory Animal Co. Ltd. (Shanghai, China) provided nude mice housed in an environment free from pathogens. The mice had ad libitum access to food and water. Animals were randomly assigned to experimental groups, with a minimum of five animals per group. For tumor xenograft studies, 50 µL of stably transfected cell solution was mixed with 50 µL of Matrigel (Corning, Catalog No. 354234) and injected subcutaneously into nude mice. When the volume of the xenograft tumor grew to 100 mm^3^, the drugs were administered. Haprolid was administered by intraperitoneal injection and MK2206-2HCI was administered by gavage. The concentration of Haprolid was 2 mg/kg. This concentration was referenced from Xing Jun et al. [5]. Haprolid was administered to nude mice on the first three days of each week and treated for a total of three weeks. The concentration of MK2206-2HCI was 240 mg/kg. It was administered three times a week for two weeks. Tumor dimensions were assessed every three days using calipers, and the formula was used to calculate the volume of the tumor: V = (Width)^2^ × Length/2.

### 2.14. Statistics

All experiments were repeated two to three times. GraphPad Prism 9.0 was used to analyze the results using Student’s *t*-test to evaluate group differences. The association between DHX9 expression status and HCC and paraneoplastic tissues was assessed using chi-square analysis conducted using SPSS 21 software. Differences were considered statistically significant when the *p*-value was <0.05 (*), <0.01 (**), <0.001 (***), or not significant (ns).

## 3. Results

### 3.1. DHX9 Was Downregulated After Haprolid Treatment of HCC Cells

**Figure 1:**
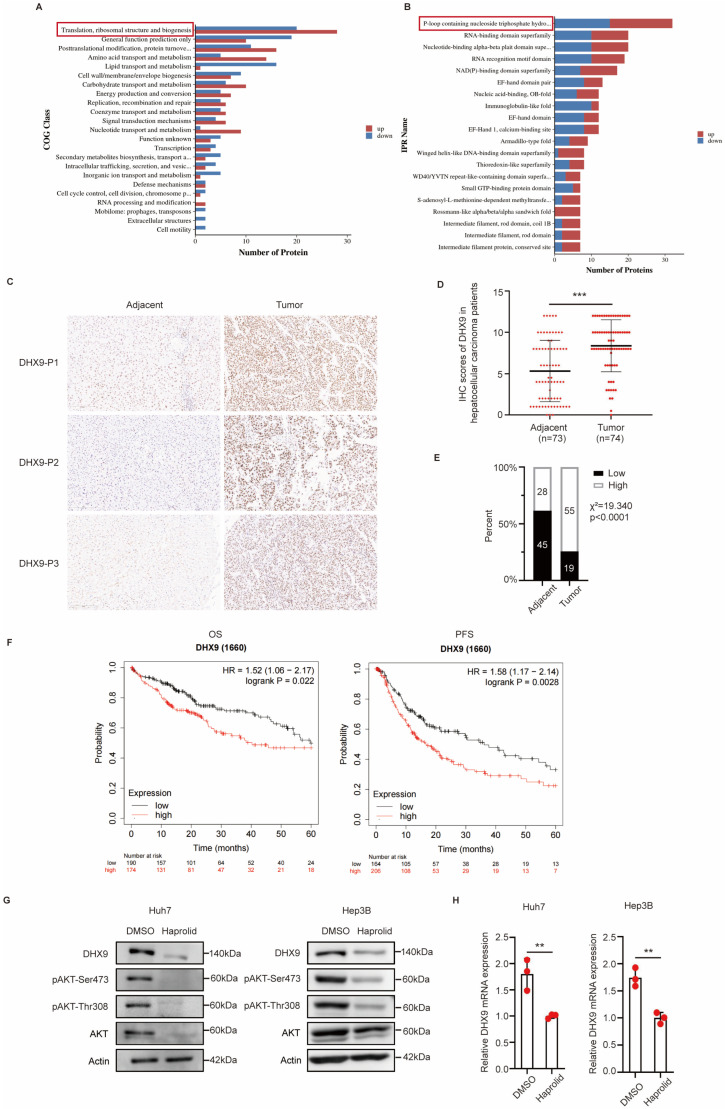
DHX9 is associated with HCC, and DHX9 was downregulated after Haprolid treatment: (**A**) COG annotation of DEPs identified by iTRAQ after Haprolid treatment. DEPs were mainly enriched in translation, ribosomal structure, and biogenesis. (**B**) IPR annotation of DEPs identified by iTRAQ after Haprolid treatment. P-loop containing nucleoside triphosphate hydrolase were the most altered structural domains after Haprolid treatment of Hep3B cells. (**C**) Representative immunohistochemical images depicting DHX9 staining in HCC tissues and the corresponding paraneoplastic tissues. Scale bars: 200 µm. (**D**) IHC scores of DHX9 in HCC tissues compared with paraneoplastic tissues of patients with HCC. (**E**) Distribution of patients with HCC based on high and low DHX9 expression in both HCC and paraneoplastic tissues. (**F**) Correlation between the expression of DHX9 and the prognosis of patients with HCC. (**G**) Relative protein expression levels of DHX9, pAKT-Ser473, pAKT-Thr308, AKT, and Actin were analyzed by Western blotting after Huh7 and Hep3B cells were treated with either DMSO or Haprolid. The uncropped blots are shown in File S1. (**H**) Relative mRNA expression levels of DHX9 were quantified by qRT-PCR after Huh7 and Hep3B cells were treated with either DMSO or Haprolid. Images were taken at 200× magnification. Scale bars: 200 µm. P1, P2, P3: Patient 1, Patient 2, Patient 3. OS: Overall survival. PFS: progression-free survival. Data are presented as mean ± SEM, ** *p* < 0.01, *** *p* < 0.001.

iTRAQ was used to profile proteomic alterations in Hep3B cells after exposure to Haprolid (6 µg/mL) for 96 h. The experimental procedure was as follows: protein acquisition, enzymatic desalting, iTRAQ labelling and fractionation, LC-MS/MS analysis, database search, and bioinformatics analysis. Significant difference was defined as log_2_FC > 1 or <−1 with adjusted *p*-value < 0.05. In total, 444 differentially expressed proteins (DEPs) were identified. Comparing the Haprolid-treated group with the control group, 205 proteins had reduced expression, and 239 proteins had elevated expression. Clusters of orthologous genes (COGs) annotation revealed that the majority of the DEPs were principally involved in biological processes related to translation, ribosomal structure, and biogenesis (Figure 1A). Meanwhile, InterPro (IPR) domain annotations indicated that a preponderance of the differentially represented domains comprised a P-loop-containing nucleoside triphosphate hydrolase (Figure 1B). Therefore, by intersecting the identified proteins involved in both the most significantly altered biological processes and the most affected domains upon Haprolid treatment, we narrowed our focus to five key proteins: EFIAI, GNL3, DHX9, EFTU, and GUF1 (Figure S1A). Prior research has established that Haprolid notably suppresses the AKT signaling pathway [5]. Subsequent protein–protein interaction analyses conducted using the STRING database revealed a close association between DHX9 and the AKT signaling pathway (Figure S1B). Additionally, based on the iTRAQ-derived differential protein scores, DHX9 was ranked in the top 20. To delineate the correlation between DHX9 and HCC, we procured tissue microarrays comprising 75 paired tumors and adjacent paracancerous tissues from HCC patients. Immunohistochemistry (IHC) was used to visualize DHX9, and the outcomes were evaluated by a pathologist. In addition, DHX9 expression was upregulated in HCC tissues compared to paracancerous tissues (Figure 1C,D). Stratifying the HCC tissues based on scores (≥8 for high expression and <8 for low expression) revealed 19 cases (25.676%) with low DHX9 expression and 55 cases (74.324%) with high DHX9 expression. Among paracancerous tissues, 45 cases (61.644%) displayed low DHX9 expression, whereas 28 cases (38.356%) exhibited high DHX9 expression (Figure 1E). Furthermore, a chi-square test revealed a significant association between HCC and DHX9 expression, with a chi-square value of 19.340 and *p* < 0.0001, indicating increased DHX9 expression in the HCC tissues. Subsequent analysis using the Kaplan–Meier Plotter demonstrated that HCC patients with high DHX9 expression experienced diminished overall survival and progression-free survival compared to those with low DHX9 expression (Figure 1F). The iTRAQ screen showed that the expression of DHX9 in Hep3B cells was reduced after Haprolid treatment, and its expression was 0.43-fold that of the control. This result was verified in subsequent experiments. A quantifiable decrease in DHX9 expression was observed at both the protein and RNA levels in Huh7 and Hep3B cells (Figure 1G,H and Figure S1C), as well as in HepG2 cells (Figure S1D,E), following a 96 h treatment with Haprolid at a concentration of 6 µg/mL. Further, we predicted that Haprolid interacts with DHX9 using molecular docking experiments (Figure S1F). The predicted binding energy of Haprolid to DHX9 is −9.017 kcal/mol, suggesting that Haprolid may interact directly with DHX9.

### 3.2. Knockdown of DHX9 Resulted in Decreased Proliferation and Migration of HCC Cells

**Figure 2:**
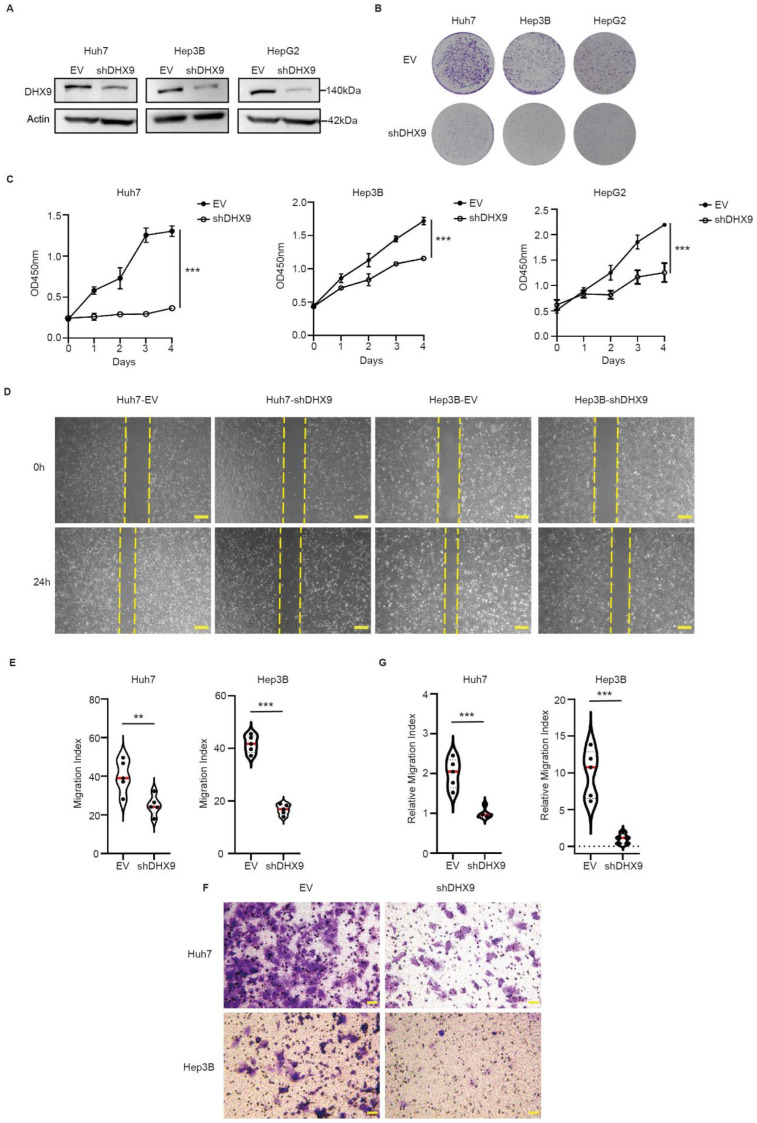
Knockdown of DHX9 effectively inhibited the proliferation and migration of HCC cells: (**A**) The knockdown efficacy of DHX9 was measured by Western blotting. The uncropped blots are shown in File S1. (**B**,**C**) Proliferation abilities were assessed after the knockdown of DHX9 using crystal violet staining and CCK-8 assays. (**D**,**E**) Migratory abilities of Huh7 and Hep3B cells post-DHX9 knockdown were evaluated using scratch assays. Images were taken at 40× magnification. Scale bars: 200 µm. (**F**,**G**) Migration capacities of Huh7 and Hep3B cells were further confirmed using transwell assays following DHX9 knockdown. Images were taken at 200× magnification. Scale bars: 50 µm. EV: empty vector. Data are presented as mean ± SEM, ** *p* < 0.01, *** *p* < 0.001.

To further explore the relationship between DHX9 and HCC, we constructed a knockdown plasmid of DHX9 (Plko.1-shDHX9). Following transfection of the DHX9 knockdown plasmid, marked downregulation of DHX9 expression was observed in the Huh7, Hep3B, and HepG2 cell lines (Figure 2A). Employing both crystal violet staining and CCK8 assays, a significant decrease in proliferative capacity was observed in DHX9-knockdown Huh7, Hep3B, and HepG2 cells relative to the EV controls (Figure 2B,C). Concurrently, a substantial reduction in the migratory capabilities of Huh7 and Hep3B cells was evidenced post-DHX9 knockdown, as assessed by the scratch (Figure 2D,E) and transwell assays (Figure 2F,G).

### 3.3. Overexpression of DHX9 Reverses Haprolid-Mediated Inhibition of Proliferation and Migration in HCC Cells

**Figure 3:**
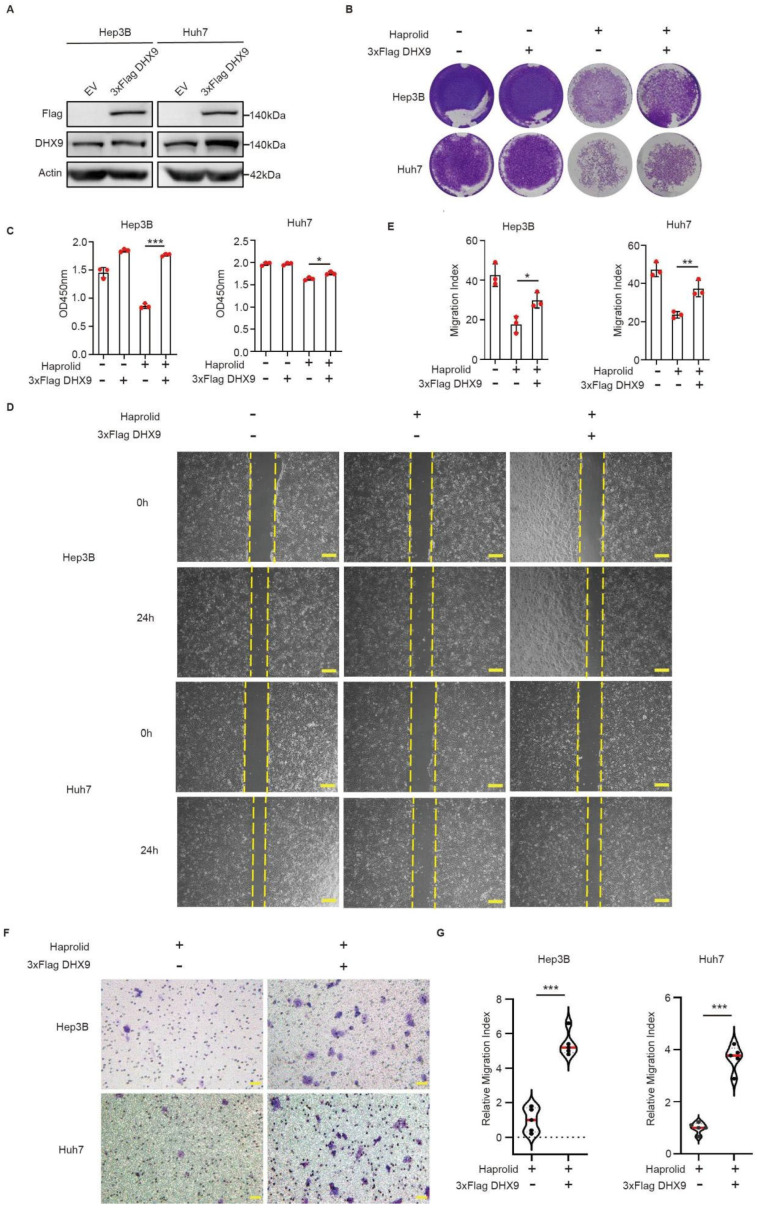
Overexpression of DHX9 restored the inhibitory effect of Haprolid on HCC cells: (**A**) The efficacy of DHX9 overexpression in Huh7 and Hep3B cells was quantified by Western blotting. The uncropped blots are shown in File S1. (**B**,**C**) The proliferative capacities of Huh7 and Hep3B cells subjected to Haprolid treatment and subsequent transfection with either EV or Flag-DHX9 were evaluated using crystal violet staining and CCK-8 assays. (**D**,**E**) The migratory abilities of Huh7 and Hep3B cells after Haprolid treatment and DHX9 overexpression were examined using scratch assays. Images were taken at 40× magnification. Scale bars: 200 µm. (**F**,**G**) The migratory abilities of Huh7 and Hep3B cells following treatment with Haprolid and transfection with either EV or Flag-DHX9 were further assessed using transwell assays. Images were taken at 200× magnification. Scale bars: 50 µm. EV: empty vector. Data are presented as mean ± SEM, * *p* < 0.05, ** *p* < 0.01, *** *p* < 0.001.

To investigate whether Haprolid functions through DHX9 to inhibit HCC growth, DHX9 was overexpressed by transfection with Plvx-3xFlag-DHX9, and the transfection efficacy was validated through Western blotting analysis (Figure 3A). After this transfection, both Huh7 and Hep3B cells exhibited increased DHX9 expression. Moreover, the inhibitory effect of Haprolid on the proliferation of these cells was negated upon DHX9 overexpression, as assessed by cellular growth assays (Figure 3B,C). Similar results were obtained in HepG2 cells (Figure S2A). Functional assays, including scratch (Figure 3D,E) and transwell migration assays (Figure 3F,G), further substantiated that DHX9 overexpression counteracted the suppressive effect of Haprolid on cellular migratory capacities.

### 3.4. DHX9 Modulates Proliferation and Migration in HCC Cells via the AKT Signaling Pathway

**Figure 4:**
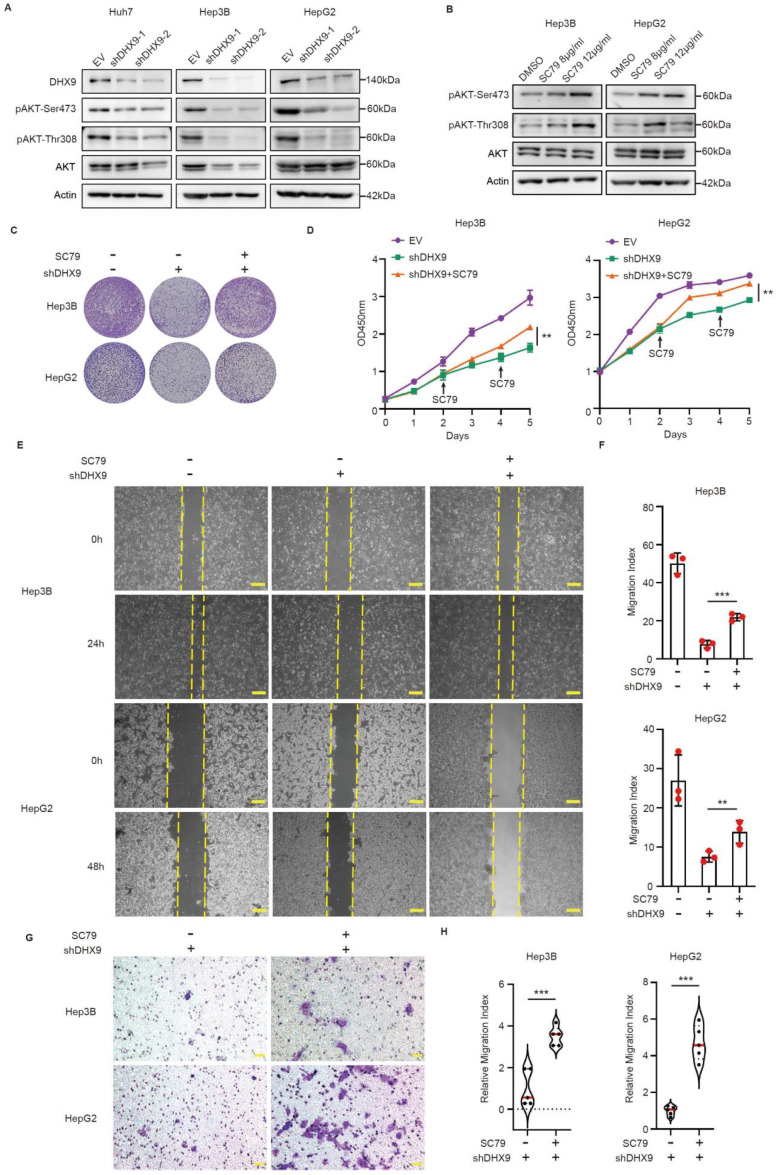
DHX9 regulated the proliferation and migration of HCC cells through the AKT signaling pathway: (**A**) The expression of DHX9, pAKT-Ser473, pAKT-Thr308, AKT, and Actin was analyzed by Western blotting after knockdown of DHX9. (**B**) Hep3B, and HepG2 cells were treated with either 8 or 12 µg/mL SC79 for 48 h, and the expression levels of pAKT-Ser473, AKT, and Actin were analyzed by Western blotting. The uncropped blots are shown in File S1. (**C**,**D**) The proliferative capacities of Hep3B and HepG2 cells subjected to SC79 treatment and subsequent transfection with either EV or plko.1-shDHX9 were evaluated using crystal violet staining and CCK-8 assays. (**E**,**F**) The migratory abilities of Hep3B and HepG2 cells, post-treatment with SC79, and subsequent transfection with either EV or plko.1-shDHX9 were assessed using scratch assays. Images were taken at 40× magnification. Scale bars: 200 µm. (**G**,**H**) The migratory abilities of Hep3B and HepG2 cells treated with SC79 and subsequently transfected with either EV or plko.1-shDHX9 were further evaluated using transwell assays. Images were taken at 200× magnification. Scale bars: 50 µm. Data are presented as mean ± SEM, ** *p* < 0.01, *** *p* < 0.001.

Previous studies have established a significant inhibitory effect of Haprolid on the AKT signaling pathway [5]. The STRING database also suggested a functional association between DHX9 and the AKT signaling pathway (Figure S1B). Based on these findings, we hypothesized that Haprolid may attenuate HCC progression by downregulating DHX9, negatively affecting the AKT signaling pathway. To evaluate this hypothesis, we assessed alterations in AKT pathway components following DHX9 knockdown in Huh7, Hep3B, and HepG2 cells using Western blotting analysis. Our data revealed that AKT pathway activity was compromised upon DHX9 knockdown (Figure 4A), whereas DHX9 overexpression led to the activation of the AKT signaling pathway (Figure S3A).

Note that SC79, an AKT activator, specifically activates the AKT signaling pathway [10]. Previous studies have demonstrated its efficacy in activating AKT in HCC cells [11,12]. After treatment with SC79, upregulation of pAKT-Ser473 and pAKT-Thr308 expression was observed by Western blotting analysis. This upregulation was concentration-dependent (Figure 4B). Crystal violet staining and CCK8 assays further revealed that DHX9 knockdown led to diminished proliferative capacity, an effect that was countered by SC79 treatment (Figure 4C,D). Additionally, the migration capability, which was reduced by DHX9 knockdown, was restored following SC79 treatment, as evidenced by the scratch (Figure 4E,F) and transwell assays (Figure 4G,H).

MK2206-2HCI, an established AKT inhibitor, has been proven to effectively attenuate the AKT signaling pathway in HCC cells [13,14]. Using Western blotting, we confirmed that MK2206-2HCI robustly suppressed pAKT-Ser473 expression (Figure S3B). Subsequent CCK8 assays revealed that, although DHX9 overexpression augmented the proliferation, this effect was mitigated by MK2206-2HCI treatment (Figure S3C). Collectively, these findings underscore DHX9’s role in modulating the proliferation and migration of HCC cells via the AKT signaling pathway.

### 3.5. Haprolid Induces Apoptosis in HCC Cells by Downregulating DHX9

**Figure 5:**
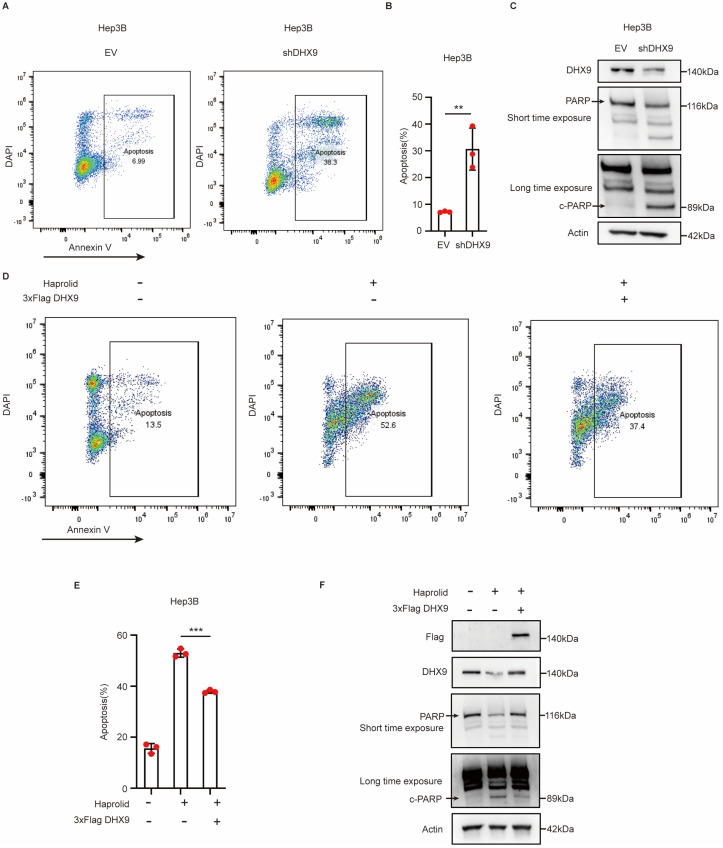
Haprolid promoted apoptosis in HCC cells by downregulating DHX9: (**A**,**B**) The apoptosis rate of Hep3B cells was measured after DHX9 knockdown by annexin V staining using flow cytometry. (**C**) Expression of PARP and c-PARP was measured after DHX9 knockdown by Western blotting. (**D**,**E**) The apoptosis rate of Hep3B cells treated with Haprolid and subsequently transfected with either EV or Flag-DHX9 was measured by annexin V staining and flow cytometry analysis. (**F**) Expression levels of PARP and c-PARP were measured by Western blotting in Hep3B cells treated with Haprolid and subsequently transfected with either EV or Flag-DHX9. The uncropped blots are shown in File S1. EV: empty vector. Data are presented as mean ± SEM, ** *p* < 0.01, *** *p* < 0.001.

A previous study established that Haprolid induces apoptosis in Hep3B cells [5]. Consequently, we sought to determine whether Haprolid mediates this apoptotic effect in Hep3B cells by downregulating DHX9. Flow cytometry using Annexin V and DAPI staining revealed an increase in apoptosis in Hep3B cells upon DHX9 knockdown (Figure 5A,B). Moreover, the cleavage of poly (ADP-ribose) polymerase (c-PARP), a hallmark of apoptosis, was assessed [15]. Western blotting analysis confirmed elevated expression of c-PARP following DHX9 knockdown in Hep3B cells (Figure 5C). Interestingly, at the same time, Haprolid treatment amplified the apoptosis rate in Hep3B cells; this effect was counteracted upon DHX9 overexpression (Figure 5D,E). Furthermore, Western blotting analyses demonstrated increased c-PARP expression in Haprolid-treated Hep3B cells; however, this elevation was suppressed in Haprolid-treated Hep3B cells that overexpressed DHX9 (Figure 5F).

### 3.6. In Vivo Validation of Haprolid-Mediated Inhibition of HCC Through DHX9 Downregulation

**Figure 6:**
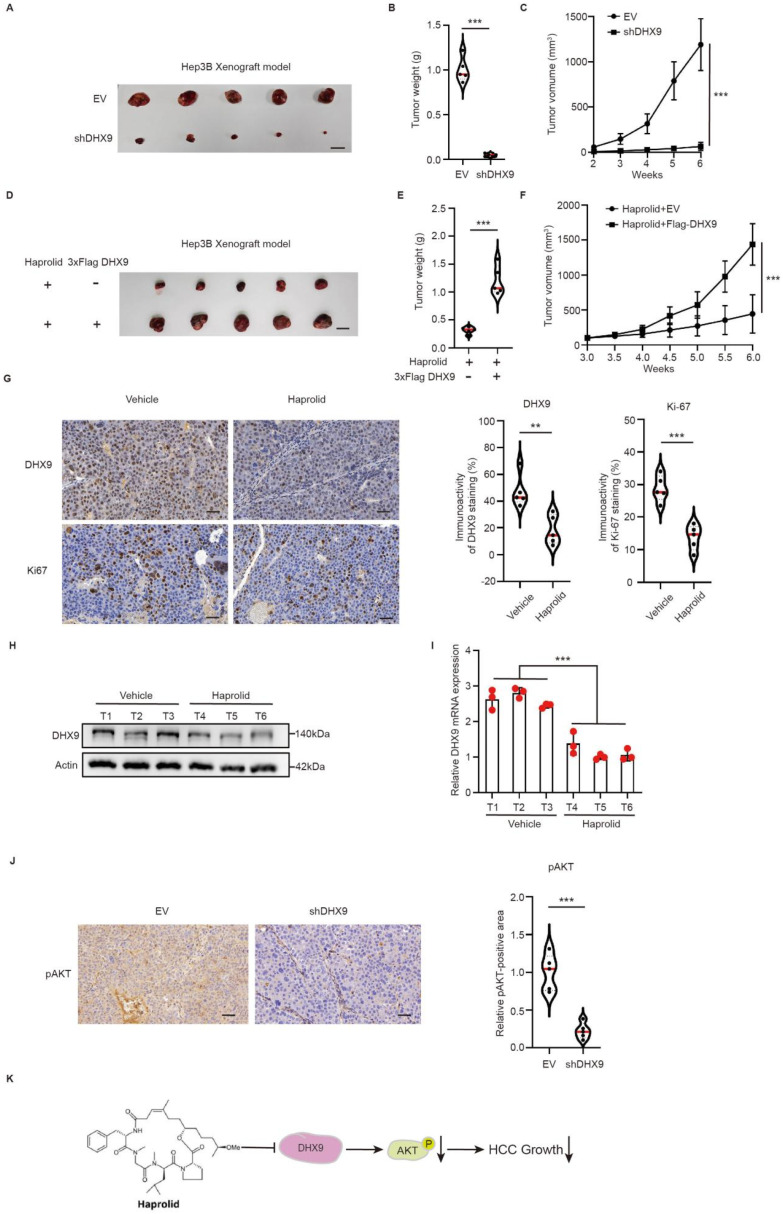
In vivo validation that Haprolid inhibits HCC growth through downregulation of DHX9. (**A**) Representative images of xenograft HCC tumors obtained from mice inoculated with either Hep3B-EV or Hep3B-shDHX9. Scale bars: 1 cm. (**B**) Comparison of tumor weights in Hep3B-EV and Hep3B-shDHX9 xenograft models. (**C**) Growth curves illustrating tumor volume progression over time in both the Hep3B-EV and Hep3B-shDHX9 groups. (**D**) Representative images of xenograft tumors from mice inoculated with Hep3B-EV or Hep3B-Flag-DHX9 cells following three-week Haprolid treatment. Scale bars: 1 cm. (**E**) Comparison of tumor weights in Haprolid-treated Hep3B-EV and Hep3B-Flag-DHX9 groups. (**F**) Tumor volume growth curves for the Hep3B-EV and Hep3B-Flag-DHX9 groups treated with Haprolid. (**G**) Representative immunohistochemical images displaying DHX9 and Ki-67 expression in xenograft HCC tumors from control and Haprolid-treated mice following three-week Haprolid treatment. Scale bars: 50 µm. (**H**,**I**) DHX9 protein and mRNA levels were analyzed in xenograft HCC tumors from control and Haprolid-treated mice. The uncropped blots are shown in File S1. (**J**) Representative immunohistochemical images displaying pAKT expression in xenograft HCC tumors from EV and shDHX9 groups. Scale bars: 50 µm. (**K**) The schematic diagram demonstrates that Haprolid inhibits the AKT signaling pathway by downregulating DHX9, thereby inhibiting HCC growth. Arrows indicate descending. EV: empty vector. Data are presented as mean ± SEM, ** *p* < 0.01, *** *p* < 0.001.

To assess the in vivo role of DHX9 in HCC progression and to determine whether Haprolid impedes HCC growth via DHX9 downregulation, we employed a subcutaneous xenograft model using Hep3B cells in nude mice. Tumor xenografts exhibited a significant reduction upon DHX9 knockdown compared with the EV control group (Figure 6A). Moreover, both tumor weight and growth trajectory indicated pronounced inhibition of tumor progression post-DHX9 knockdown (Figure 6B,C).

Additionally, we established a xenograft model using nude mice implanted with Hep3B cells overexpressing DHX9 alongside an EV xenograft control. Once xenograft tumors achieved an approximate size of 100 mm^3^, we initiated treatment with Haprolid at a dose of 2 mg/kg. Following a three-week treatment period, xenografts overexpressing DHX9 were larger than those in the control group (Figure 6D). Consistently, the tumor weight and growth trajectories suggested that xenografts overexpressing DHX9 demonstrated enhanced resistance to Haprolid intervention (Figure 6E,F).

Mice bearing Hep3B tumor xenografts were randomly assigned to two cohorts. Once tumors approached an approximate size of 100 mm^3^, one cohort was administered Haprolid at a dosage of 2 mg/kg. Following three weeks of intervention, Haprolid-treated xenografts presented with reduced dimensions (Figure S4A) and diminished mass (Figure S4B) relative to untreated controls. IHC analyses of mice in the Haprolid cohort revealed attenuated expression of both DHX9 and Ki-67 (Figure 6G). Furthermore, assessment of DHX9 expression in tumors via Western blotting and qPCR demonstrated its downregulation in the presence of Haprolid (Figure 6H,I). Collectively, these findings underscore the pivotal role of DHX9 in HCC tumorigenesis in vivo and establish that Haprolid impedes HCC progression by attenuating DHX9.

The relationship between DHX9 and the AKT pathway in vivo was further investigated. Tumor tissues from EV and shDHX9 groups were stained for pAKT, and the results showed that DHX9 knockdown in vivo resulted in downregulation of pAKT expression (Figure 6J). Rescue experiments were performed with MK2206-2HCI (AKT inhibitor). Compared to the EV group, the tumors in the DHX9 overexpression group were larger. The mice in the DHX9-overexpressing group were then treated with MK2206-2HCI, which inhibited tumor growth (Figure S4C,D). In summary, our data corroborate that Haprolid inhibits HCC proliferation by attenuating the AKT signaling pathway via DHX9 downregulation (Figure 6K).

## 4. Discussion

Haprolid, an emergent small-molecule compound, has exhibited notable efficacy in curtailing HCC growth both in vivo and in vitro. Notably, Haprolid displayed selective cytotoxicity in a spectrum of tumor cells. The mechanisms underlying this selective toxicity remain to be fully elucidated. However, they could be associated with the specific molecular targets of the compound. Preliminary investigations suggest that Haprolid mitigates HCC growth by modulating the AKT signaling pathway. Nevertheless, the intricate dynamics by which Haprolid impedes HCC growth and its specific interactions within the AKT pathway warrant further exploration. Thus, a deeper understanding of the mechanisms is pivotal for optimizing the translational potential of Haprolid in clinical settings.

Employing iTRAQ proteomic techniques, we identified a distinctive expression pattern of DHX9, an ATP-dependent RNA helicase, following treatment with Haprolid. DHX9 is recognized for its involvement in essential gene regulatory processes, including DNA replication, transcription, RNA modification, transport, and genome integrity maintenance [9]. During transcription, DHX9 binds to RNA Pol II and ensures efficient transcription by unwinding the R-loop. During replication, DHX9 recruits BRCA1 to sites of transcription/replication conflict, allowing it to repair stalled replication forks. In addition, DHX9 recruits BRCA1 to sites of dsDNA breaks, allowing it to initiate homologous end repair and maintain genomic stability [7]. DHX9 plays diverse roles in distinct tumor types. Notably, in lung, breast, renal, and colorectal cancer, and glioma, DHX9 has been associated with promoting cell proliferation and migration [16,17,18]. Conversely, in papillary thyroid cancer, DHX9 exhibits an opposing effect, dampening cell proliferation and migration [19]. The contextual variability in DHX9’s functional role underscores its tumor-specific tumorigenic properties. Numerous studies have explored the interplay between DHX9 with circular RNA (circRNAs) and long non-coding RNA (lncRNAs), implicating DHX9 in regulatory networks governing tumorigenesis [20,21,22,23]. Under UV irradiation, DHX9 forms DHX9 stress granules, which play a unique role in tumors [24]. Additionally, Liu et al. highlighted the correlation between DHX9 and tumor-associated macrophage infiltration [18]. Targeting DHX9 triggers tumor-intrinsic interferon response and replication stress in small-cell lung cancer. Inhibition of DHX9 induces cellular dsRNA accumulation and triggers tumor intrinsic innate immunity [25]. Contemporary research has further emphasized DHX9’s pivotal role in orchestrating antiviral immunity [26]. Whether Haprolid can influence the immune microenvironment of HCC through DHX9 is not known. This needs to be further explored.

In HCC tissues, conspicuous upregulation of DHX9 expression has been observed, which correlates with the progression of tumor stages [8]. Wang Y and F. Shi reported that depletion of DHX9 inhibits HCC growth [8,17]. Bioinformatics analyses conducted by Shan Y and Chen D et al. substantiated DHX9 as a potential prognostic marker for HCC [27,28]. In vitro experiments further revealed that DHX9 knockdown enhances radiosensitivity in HCC by increasing DNA damage [29]. Additionally, Wu K suggested that DHX9 participates in HBV replication in HCC [30]. Targeting DHX9 alongside AURKB has been reported to promote HCC progression via activation of the PI3K/AKT/mTOR pathway [31]. Our data indicate that Haprolid impedes HCC growth through the modulation of DHX9, and this suppressive effect can be counteracted by DHX9 overexpression.

The AKT pathway orchestrates critical biological processes, including cell survival, proliferation, growth, glycogen metabolism, angiogenesis, and metastasis [32]. The PI3K/AKT/mTOR cascade plays a pivotal role in HCC tumorigenesis [33]. Although numerous efforts have been devoted to devising small-molecule inhibitors targeting this axis, most have not yielded the anticipated outcomes [34]. Consequently, our study aimed to elucidate the molecular intricacies governing the modulation of the AKT pathway by Haprolid. To date, no direct association between DHX9 and the AKT signaling pathway in HCC has been established. Notably, previous studies have indicated that DHX9 knockdown in breast cancer attenuates the AKT pathway, whereas its overexpression amplifies it [35,36]. Conversely, in papillary thyroid carcinoma, DHX9 silencing activates the AKT pathway [19]. Knockdown of DHX9 in myelodysplastic syndromes inactivates PI3K-AKT signaling [37]. Such incongruities underscore that DHX9’s interactions with the AKT pathway might be tumor-type-specific, potentially rationalizing DHX9’s disparate roles across malignancies. Our findings corroborate the hypothesis that Haprolid impedes the AKT pathway chiefly via DHX9 downregulation. However, given the intricate web of interactions within the AKT pathway, intertwined with a multitude of regulatory molecules, the DHX9-AKT axis might not adhere to a simplistic, unidirectional paradigm. It is conceivable that DHX9 exerts its influence on the AKT pathway by modulating an array of regulators, introducing an intricate layer of regulation that remains to be deciphered.

While the findings of this study provide a foundational understanding of the molecular mechanism underlying Haprolid’s effects on HCC, it is important to acknowledge its limitations. The effect of DHX9 and its regulation by Haprolid may vary across different HCC subtypes, including those defined by genetic mutations, immune profiles, or metabolic characteristics. Subtype-specific differences could influence the efficacy of Haprolid and its potential clinical applications. Future studies are warranted to clarify the potential subtype-specific roles of DHX9 and the effects of Haprolid across diverse HCC subtypes. These investigations could provide a more precise understanding of the compound’s therapeutic potential and inform its application in tailored treatment strategies. Although current treatment paradigms for advanced HCC increasingly emphasize immunotherapy—particularly immune checkpoint inhibitors, such as PD-1/PD-L1 and CTLA-4 blockade [38,39]—our study did not investigate whether Haprolid exerts immunomodulatory effects. The complex immune microenvironment in HCC plays a pivotal role in therapy response, and examining Haprolid’s influence on tumor-infiltrating immune cells could provide additional therapeutic insights. Future research should evaluate potential synergy between Haprolid and ICIs, as well as its impact on immune cell function and cytokine profiles. In addition to the demonstrated efficacy of Haprolid in inhibiting DHX9 and suppressing AKT signaling, evaluating its potential synergy with existing HCC treatments, such as sorafenib, would be an important next step. Combination therapies may provide enhanced tumor suppression through complementary mechanisms, potentially reducing dose requirements and minimizing toxicity. Future studies should investigate the pharmacodynamic interactions between Haprolid and standard-of-care agents to better define its clinical application scenarios and optimize therapeutic outcomes in HCC. In addition to this, this study did not focus on the pharmacological properties (solubility, metabolic pathways, and toxicity) of Haprolid, which can help us to further evaluate the potential of Haprolid for clinical applications. Despite these limitations, our findings establish a strong foundation for Haprolid as a promising candidate for targeted molecular therapy in HCC.

## 5. Conclusions

In summary, the present study demonstrated that Haprolid inhibits the AKT signaling pathway by downregulating DHX9, inhibiting HCC growth. These findings will help Haprolid enter clinical trials and may provide a new direction for developing targeted therapies for HCC.

## Data Availability

Dataset available on request from the authors.

## Supplementary Materials

The following supporting information can be downloaded at: https://www.mdpi.com/article/10.3390/cancers17030443/s1, Figure S1. DHX9 played a role in Haprolid inhibition of HCC growth; Figure S2. DHX9 overexpression reversed the inhibitory effect of Haprolid in HepG2 cells; Figure S3. Haprolid inhibited the AKT signaling pathway by regulating DHX9; Figure S4. Haprolid inhibited xenograft tumors growth in vivo; File S1: Full pictures of the Western blots.
