# Supplementary material for: Targeting Hepatocellular Carcinoma Growth: Haprolid’s Inhibition of AKT Signaling Through DExH-Box Helicase 9 Downregulation
Source: Cancers (Basel). 2025 Jan 28;17(3):443. doi: 10.3390/cancers17030443 (PMC11816161; doi:10.3390/cancers17030443)
Supplement: Supplementary file 1 [file cancers-17-00443-s001.zip › Figure S1.pdf]

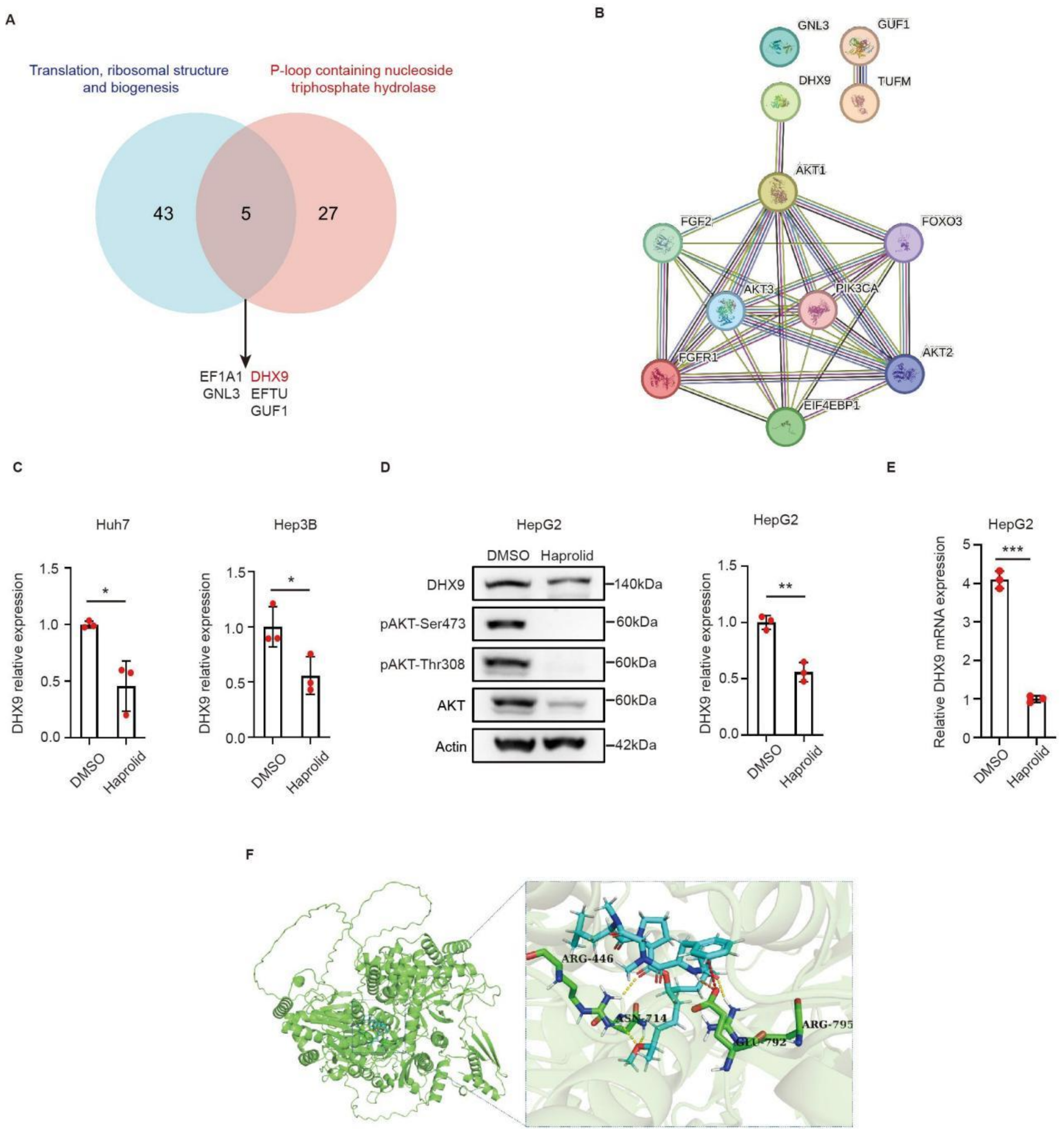

Supplemental Figure S1. DHX9 played a role in Haprolid inhibition of HCC growth

A, Venn diagram illustrating the overlap among translation, ribosomal structure, biogenesis, and P-loop containing nucleoside triphosphate hydrolase. B, STRING database representation elucidating the interactions between the AKT signaling pathway and proteins DHX9, GUF1, GNL3, and TUFM. C, Grey level statistics of DHX9 expression after Haprolid treatment in Fig. 1G. D, Western blotting analysis of DHX9, pAKT-Ser473, pAKT-Thr308, AKT, and actin expression in HepG2 cells after treatment with either DMSO or Haprolid. E, Relative mRNA expression levels of DHX9 were quantified by qRT-PCR after HepG2 cells were treated with either DMSO or Haprolid. F, Molecular docking experiment predicted that Haprolid interacts with DHX9.
