# Supplementary material for: Targeting Hepatocellular Carcinoma Growth: Haprolid’s Inhibition of AKT Signaling Through DExH-Box Helicase 9 Downregulation
Source: Cancers (Basel). 2025 Jan 28;17(3):443. doi: 10.3390/cancers17030443 (PMC11816161; doi:10.3390/cancers17030443)
Supplement: Supplementary file 1 [file cancers-17-00443-s001.zip › Figure S2.pdf]

A

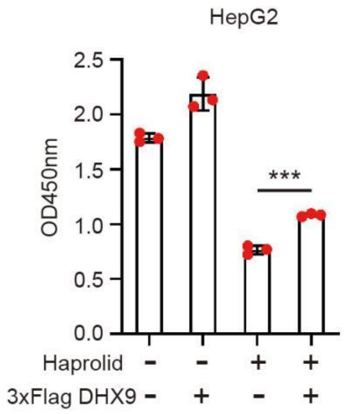

Supplemental Figure S2. DHX9 overexpression reversed the inhibitory effect of Haprolid in HepG2 cells

A, The proliferative capacities of HepG2 cells subjected to Haprolid treatment and subsequent transfection with either EV or Flag-DHX9 were evaluated using CCK-8 assays.
