# Supplementary material for: Targeting Hepatocellular Carcinoma Growth: Haprolid’s Inhibition of AKT Signaling Through DExH-Box Helicase 9 Downregulation
Source: Cancers (Basel). 2025 Jan 28;17(3):443. doi: 10.3390/cancers17030443 (PMC11816161; doi:10.3390/cancers17030443)
Supplement: Supplementary file 1 [file cancers-17-00443-s001.zip › Figure S3.pdf]

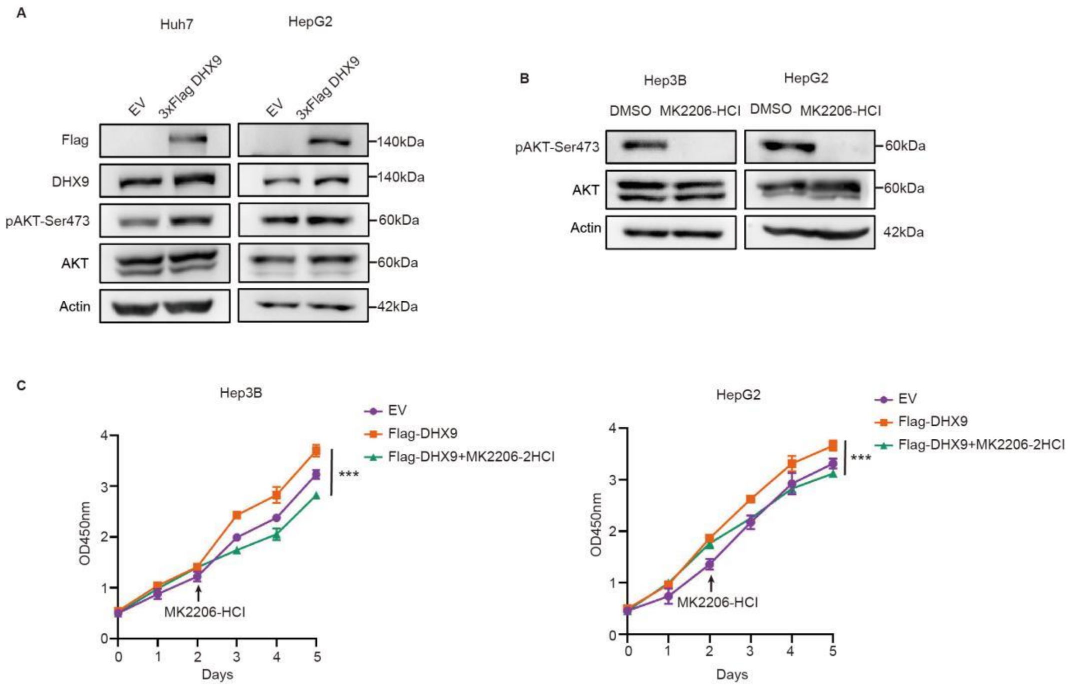


**Supplemental Figure S3. Haprolid inhibited the AKT signaling pathway by regulating DHX9**

A, The expression of Flag, DHX9, pAKT-Ser473, AKT and Actin was analyzed by western blotting after overexpression of DHX9 in Huh7 and HepG2 cells. B, Hep3B and HepG2 cells were treated with 5 µmol/L MK2206-2HCl for 96 h, and the expression levels of pAKT-Ser473, AKT, and Actin were analyzed by western blotting. C, The proliferative capacities of Hep3B and HepG2 cells subjected to MK2206-2HCl treatment and subsequent transfection with either EV or Flag-DHX9 were evaluated using CCK-8 assays. EV: empty vector.
