# Supplementary material for: Targeting Hepatocellular Carcinoma Growth: Haprolid’s Inhibition of AKT Signaling Through DExH-Box Helicase 9 Downregulation
Source: Cancers (Basel). 2025 Jan 28;17(3):443. doi: 10.3390/cancers17030443 (PMC11816161; doi:10.3390/cancers17030443)
Supplement: Supplementary file 1 [file cancers-17-00443-s001.zip › Figure S4.pdf]

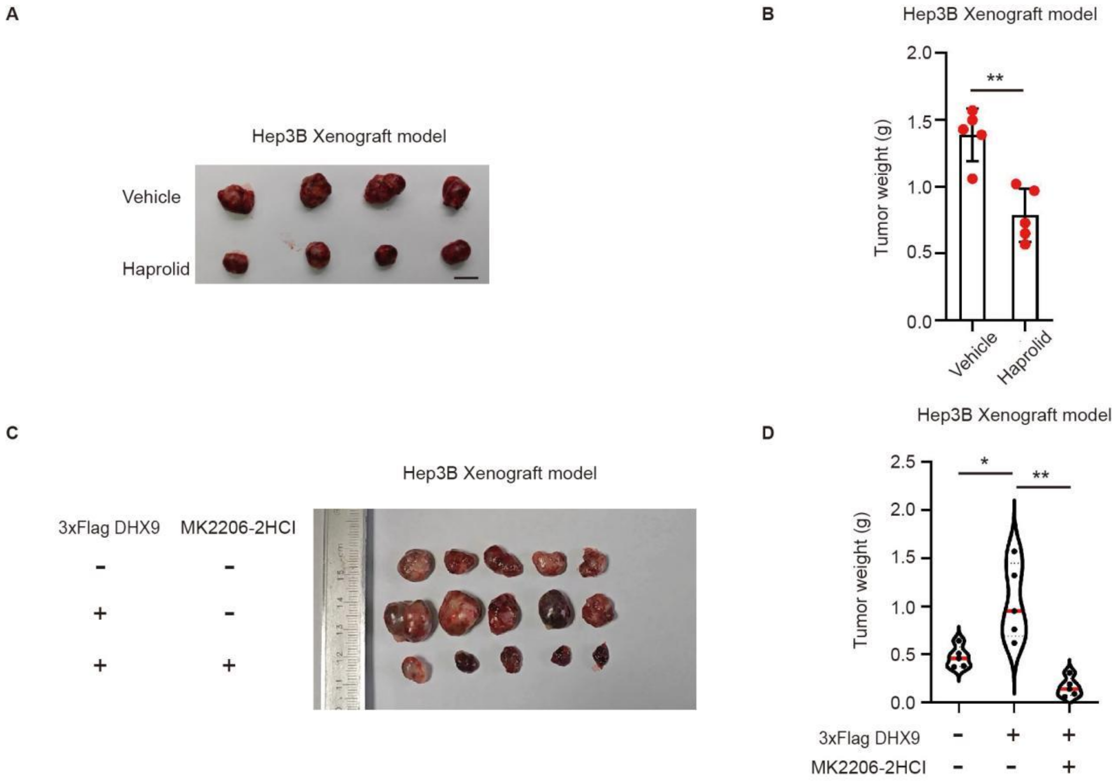

Supplemental Figure S4. Haprolid inhibited xenograft tumors growth in vivo

A, Representative images of xenograft tumors from mice treated with Haprolid or vechicle. Scale bars: 1 cm. B, Comparison of tumor weights in xenograft models treated with vehicle and Haprolid. C, Representative images of xenograft tumors from mice inoculated with Hep3B-EV, Hep3B-Flag-DHX9 cells and Hep3B-Flag-DHX9 cells following MK2206-2HCl treatment. D, Comparison of tumor weights in Hep3B-EV, Hep3B-Flag-DHX9 and MK2206-2HCl treated Hep3B-Flag-DHX9 groups.
