# Supplementary figures and images for: Targeting Hepatocellular Carcinoma Growth: Haprolid’s Inhibition of AKT Signaling Through DExH-Box Helicase 9 Downregulation
Source: Cancers (Basel). 2025 Jan 28;17(3):443. doi: 10.3390/cancers17030443 (PMC11816161; doi:10.3390/cancers17030443)

Figure1E

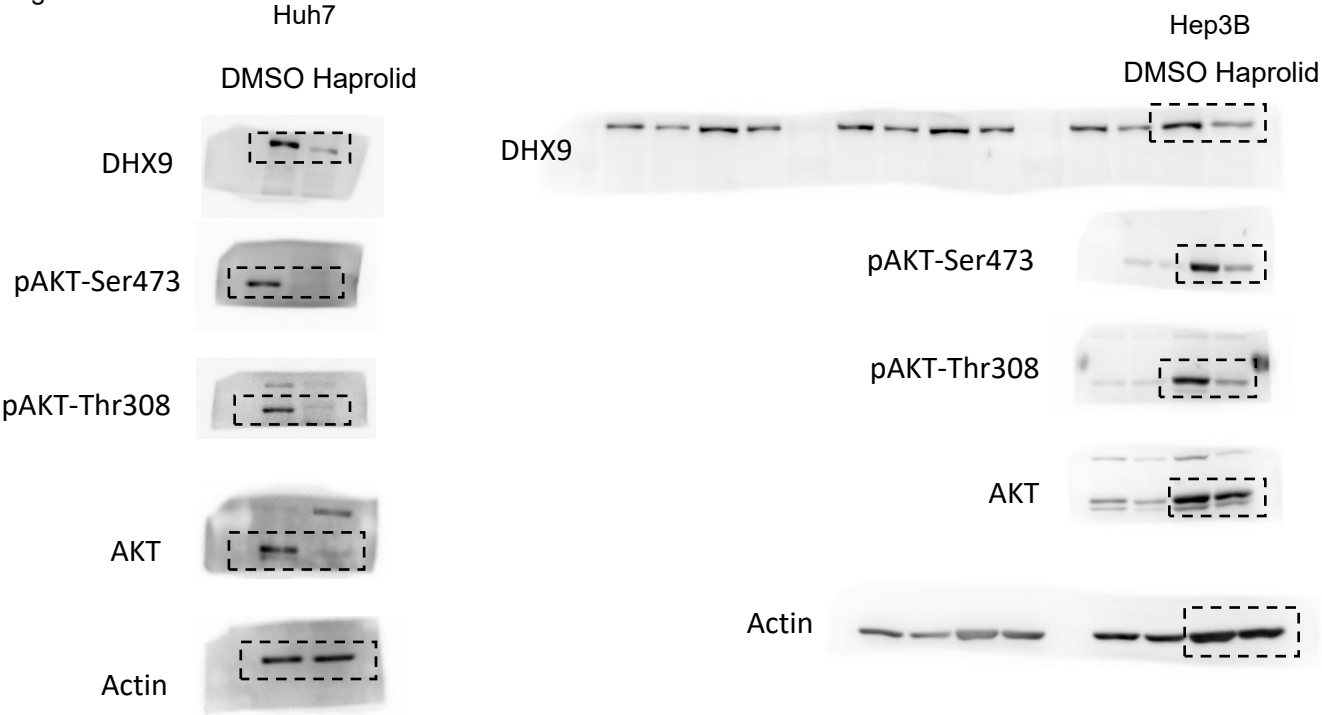

Figure2A

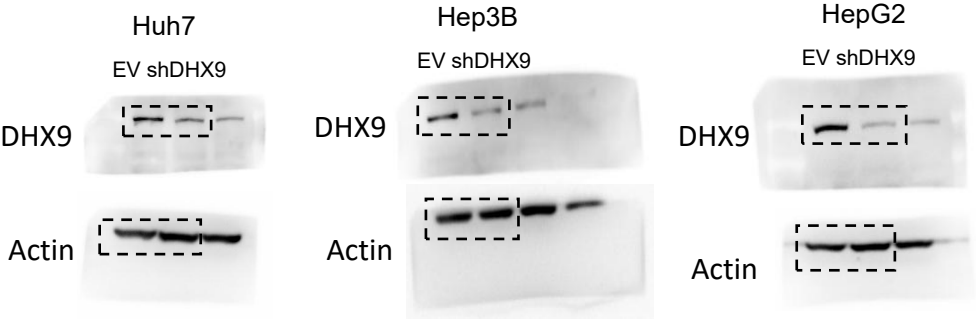

Figure3A

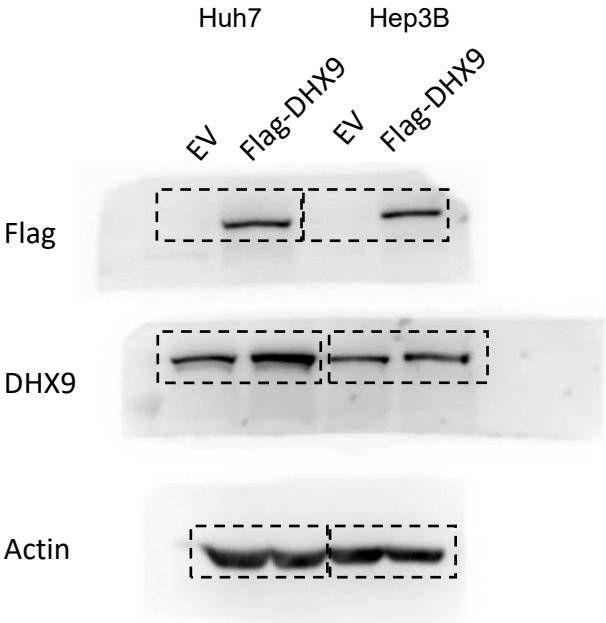

Figure4A

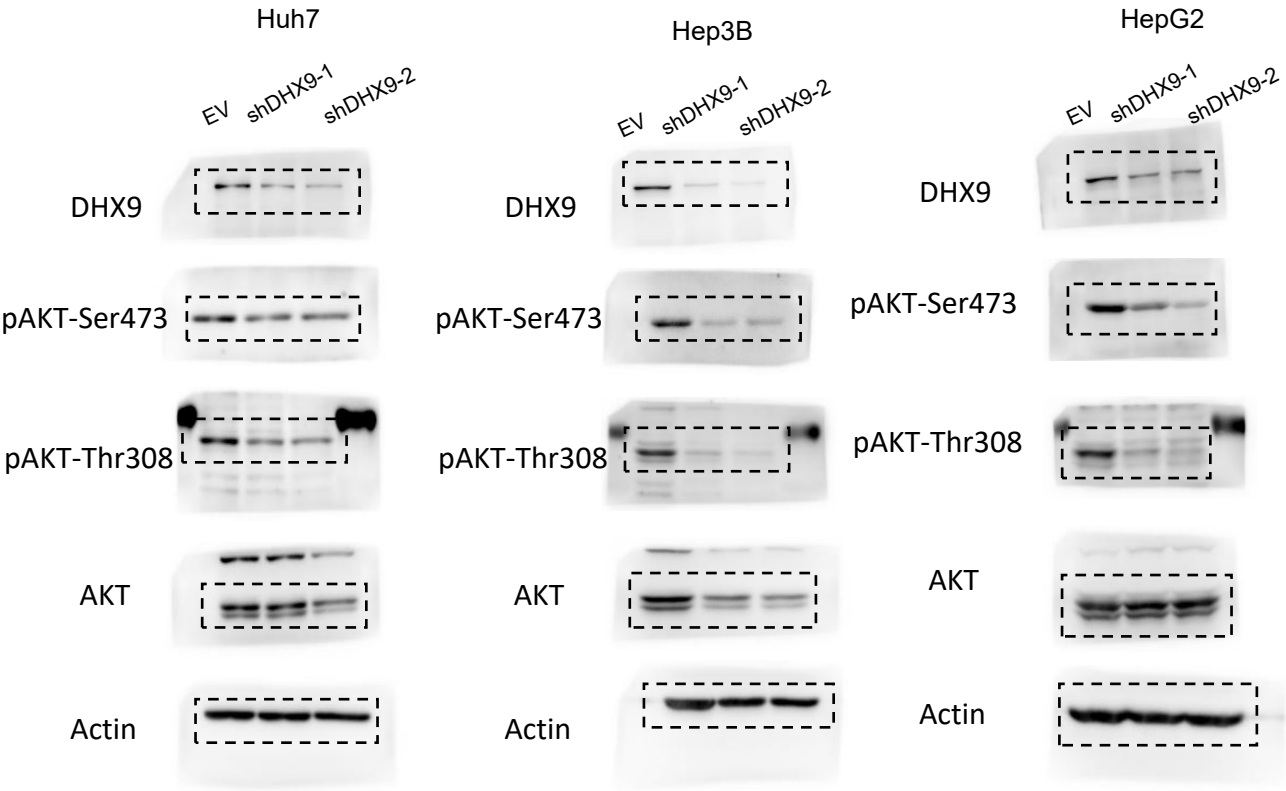

Figure4B

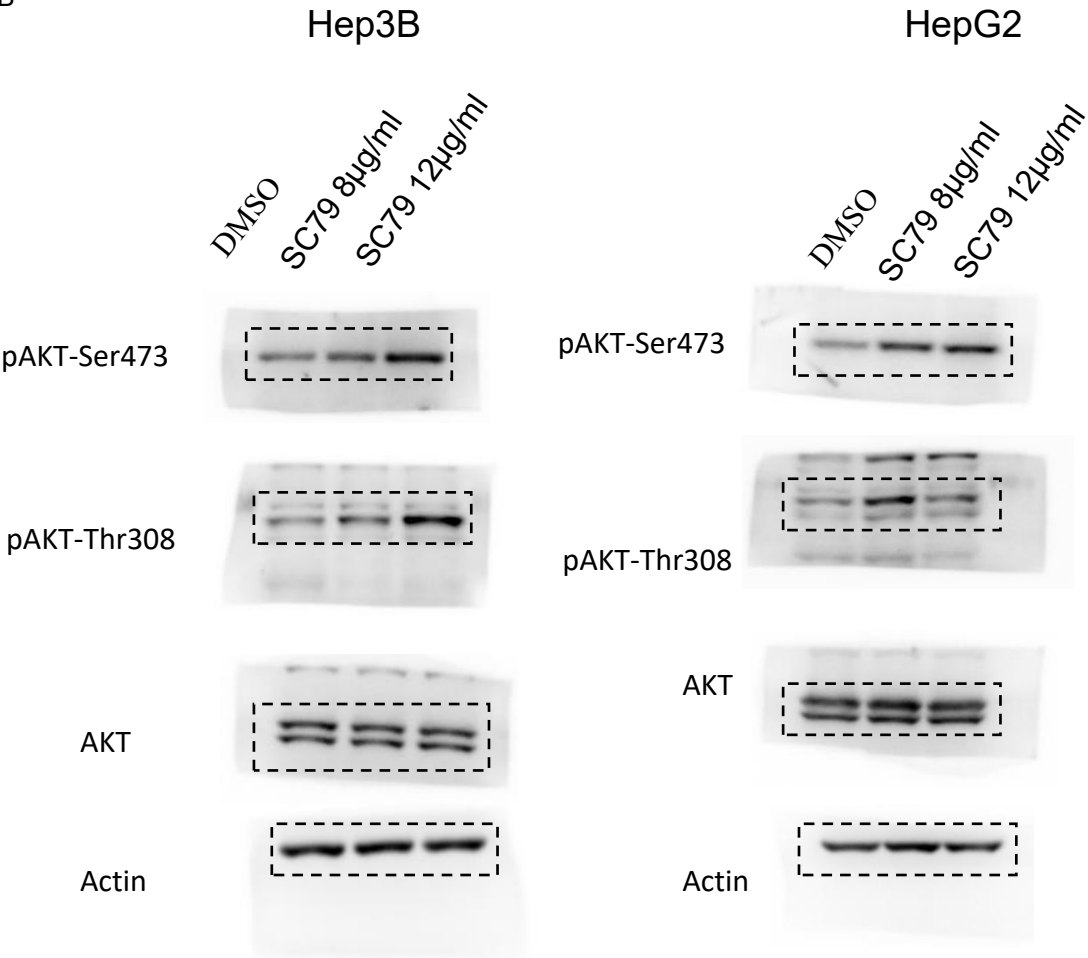

Figure5C

Figure5F

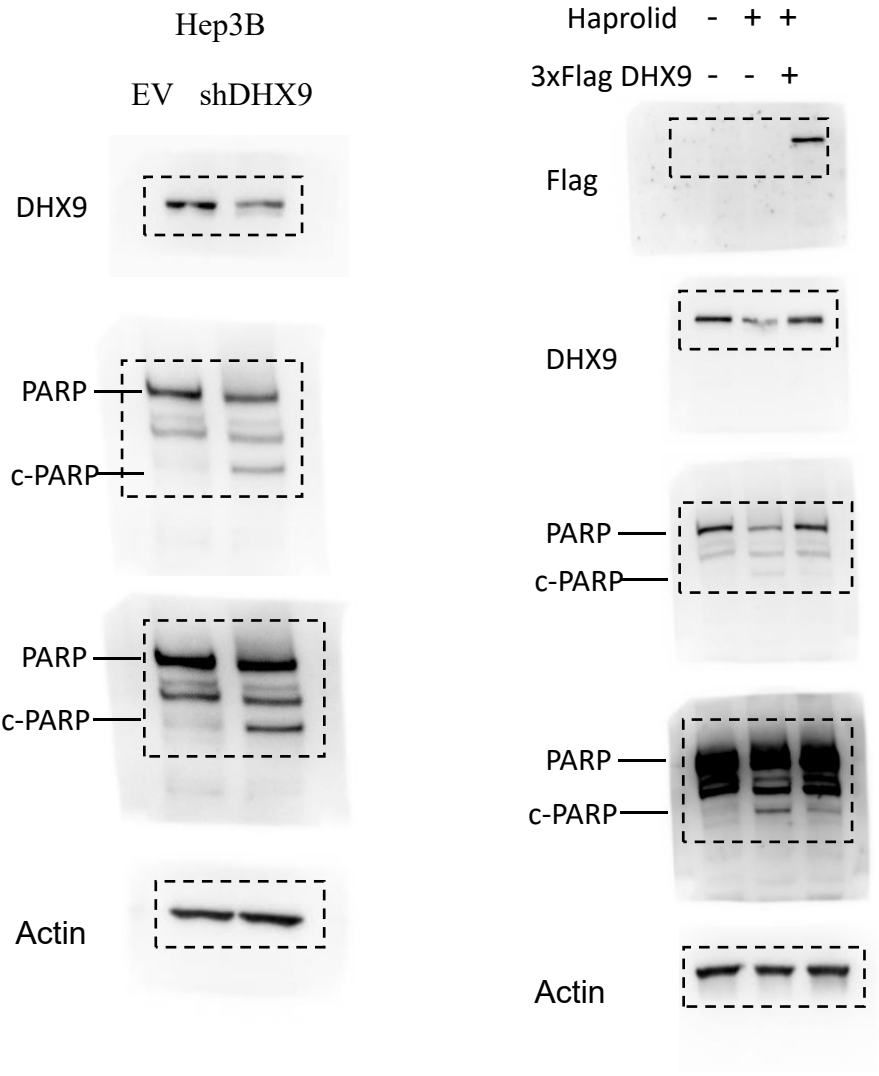

Figure6H

Vehicle      Haprolid

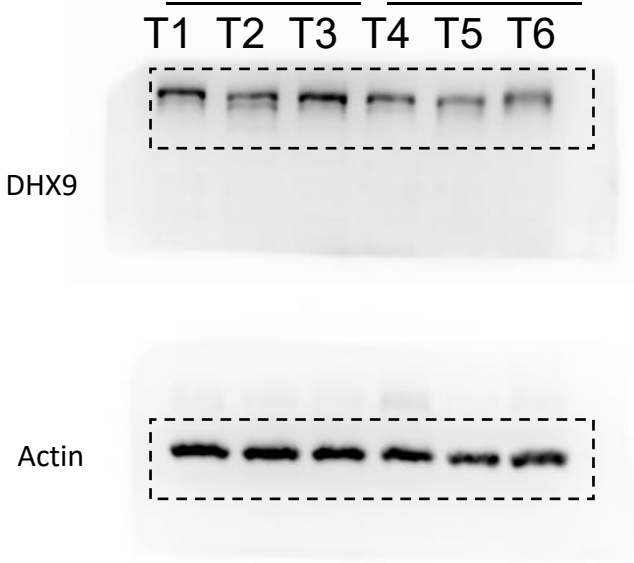

Supplement: Supplementary file 1 [file cancers-17-00443-s001.zip › File S1.pdf]
